# SHILO, a novel dual imaging approach for simultaneous HI-/LOw temporal (Low-/Hi-spatial) resolution imaging for vascular dynamic contrast enhanced cardiovascular magnetic resonance: numerical simulations and feasibility in the carotid arteries

**DOI:** 10.1186/1532-429X-15-42

**Published:** 2013-05-24

**Authors:** Claudia Calcagno, Philip M Robson, Sarayu Ramachandran, Venkatesh Mani, Melanie Kotys-Traughber, Matthew Cham, Stefan E Fischer, Zahi A Fayad

**Affiliations:** 1 Translational and Molecular Imaging Institute, Imaging Science Laboratories, Icahn School of Medicine at Mount Sinai, One Gustave L. Levy Place, Box 1234, New York, NY 10029, USA; 2 Department of Radiology, Icahn School of Medicine at Mount Sinai, One Gustave L. Levy Place, New York, NY 10029, USA; 3 MR Clinical Science, Philips Healthcare, 595 Miner Road, Cleveland, OH 44143, USA

**Keywords:** Atherosclerosis, Neovascularization, Perfusion/permeability

## Abstract

**Background:**

Dynamic contrast enhanced (DCE) cardiovascular magnetic resonance (CMR) is increasingly used to quantify microvessels and permeability in atherosclerosis. Accurate quantification depends on reliable sampling of both vessel wall (VW) uptake and contrast agent dynamic in the blood plasma (the so called arterial input function, AIF). This poses specific challenges in terms of spatial/temporal resolution and matched dynamic MR signal range, which are suboptimal in current vascular DCE-CMR protocols. In this study we describe a novel dual-imaging approach, which allows acquiring simultaneously AIF and VW images using different spatial/temporal resolution and optimizes imaging parameters for the two compartments. We refer to this new acquisition as SHILO, Simultaneous HI-/LOw-temporal (low-/hi-spatial) resolution DCE-imaging.

**Methods:**

In SHILO, the acquisition of low spatial resolution single-shot AIF images is interleaved with segments of higher spatial resolution images of the VW. This allows sampling the AIF and VW with different spatial/temporal resolution and acquisition parameters, at independent spatial locations. We show the adequacy of this temporal sampling scheme by using numerical simulations. Following, we validate the MR signal of SHILO against a standard 2D spoiled gradient recalled echo (SPGR) acquisition with *in vitro* and *in vivo* experiments. Finally, we show feasibility of using SHILO imaging in subjects with carotid atherosclerosis.

**Results:**

Our simulations confirmed the superiority of the SHILO temporal sampling scheme over conventional strategies that sample AIF and tissue curves at the same time resolution. Both the median relative errors and standard deviation of absolute parameter values were lower for the SHILO than for conventional sampling schemes. We showed equivalency of the SHILO signal and conventional 2D SPGR imaging, using both *in vitro* phantom experiments (R^2^ =0.99) and *in vivo* acquisitions (R^2^ =0.95). Finally, we showed feasibility of using the newly developed SHILO sequence to acquire DCE-CMR data in subjects with carotid atherosclerosis to calculate plaque perfusion indices.

**Conclusions:**

We successfully demonstrate the feasibility of using the newly developed SHILO dual-imaging technique for simultaneous AIF and VW imaging in DCE-CMR of atherosclerosis. Our initial results are promising and warrant further investigation of this technique in wider studies measuring kinetic parameters of plaque neovascularization with validation against gold standard techniques.

## Background

Inflammation accompanied by the proliferation of adventitial microvessels and intra-plaque neovessels is an important hallmark of vulnerable atherosclerotic plaques, at high risk of causing severe clinical events [1]. Dynamic Contrast Enhanced (DCE) CMR is a well-established non-invasive method used to quantify the extent and properties of tissue microvasculature [2-4]. In recent years DCE-CMR has been applied to quantify neovascularization in atherosclerosis [5,6] and to track its changes after the application of anti-atherosclerotic therapies [7-9]. Nowadays DCE-CMR is used as a novel endpoint in pre-clinical and clinical trials testing the efficacy of anti-atherosclerotic drugs [10,11].

DCE-CMR of atherosclerosis currently suffers from inherent limitations. Reliable estimation of plaque perfusion indices with DCE-CMR requires accurate sampling of both the blood plasma (arterial input function, AIF) and vessel wall (VW) enhancement curves [12,13]. Accurate AIF sampling requires acquiring data with high temporal resolution, which is very challenging when simultaneously imaging tissues that require high spatial resolution, such as atherosclerotic plaques [5,6,14]. To maintain acceptable spatial and temporal resolution, few axial slices are usually imaged, therefore sacrificing coverage along a vascular bed [5,6]. Moreover, AIF and VW enhancements reach very different peak contrast agent concentrations which makes it difficult to optimize imaging parameters [15] for both compartments in the same acquisition. These limitations may result in inadequate characterization of plaque neovessels and may impact the significance of DCE-CMR findings.

Recent studies have shown by numerical simulations that if the AIF is acquired with a substantially higher temporal resolution (≈1 s) than is necessary for the tissue response, then the accuracy of perfusion parameters is higher than if both curves were sampled at the same slower temporal resolution [16,17]. Many k-space sharing techniques, such as TRICKS [18-20], CURE [21], keyhole [22-24], and other segmented radial [25,26]- or spiral [27]-trajectory strategies, which are densely sampled at the center of k-space, allow for increased temporal resolution in a low-spatial resolution AIF image while still acquiring time-course tissue data at higher spatial resolutions. However, these approaches still do not address the fact that AIF and tissue response need to be acquired with different dynamic signal range.

Dual bolus [28-35] and dual imaging techniques [15,36-40] can address this additional need for different dynamic signal range for AIF and VW. Dual bolus techniques allow acquiring AIF and VW images with different spatial and temporal resolutions, spatial locations and volumetric coverage. They provide different dynamic signal range for AIF and tissue by using two injections of contrast media: the first low dose injection is used for AIF acquisition, while the second full dose injection is used to acquire the tissue enhancement curves. However, to achieve good correspondence, either the two injections have to be matched in terms of volumes and injection rates [41], or appropriate steps have to be followed while processing the data [42]. Both approaches are either not practical in a clinical setting, or render data analysis more complicated, with a higher degree of uncertainty.

Differently from dual-bolus, dual-imaging techniques can acquire AIF and VW data with different imaging parameters simultaneously, in the same scan, after only one injection of the full dose required for imaging uptake in the target tissue, such as the vessel wall [5,15,36-38,40]. This eliminates the need for dilution and matching of volume and rate of separate injections and allows measuring the AIF during uptake in the tissue of interest. In addition, since it requires only a single injection, this approach is ideal for ease of workflow in a clinical setting. However, in our knowledge the currently available dual imaging approaches do not allow acquiring images with different temporal and spatial resolution, spatial location and volumetric coverage. This would be particularly useful for tissues requiring imaging with high spatial resolution, such as atherosclerotic plaques.

Here we describe a novel dual-imaging method to address the described limitations of DCE-CMR of atherosclerosis. We name the new sequence SHILO (Simultaneous HI-/LOw-Temporal (Low-/Hi-Spatial) Resolution DCE-imaging). Being a dual imaging approach, both AIF and VW images can be acquired with their optimal dynamic signal range. Differently from previous dual imaging approaches this new sequence allows the acquisition of high temporal/low spatial resolution AIF images and low temporal/high spatial resolution tissue images with different temporal and spatial resolution, imaging parameters and slice location. The high temporal/low spatial resolution AIF images allow sampling the time course of the contrast agent concentration in the blood plasma at a sufficient rate. The low temporal/high spatial resolution tissue images allow sampling the slower uptake in the vessel wall with sufficient spatial resolution to capture plaques heterogeneity. Since these features are implemented in a dual-imaging approach, optimal AIF and VW curves can be obtained using only one injection of contrast agent, which is most practical in a clinical setting.

In the first part of this manuscript we present a description of the new SHILO sequence. Secondly, we perform numerical simulations to demonstrate that our chosen temporal sampling strategy (high temporal/low spatial resolution AIF interleaved with low temporal/high spatial resolution VW images) allows more accurate estimates of plaque perfusion, with respect to sampling both tissues at the same, slower rate. Thirdly, we present phantom and *in vivo* validation experiments, to demonstrate equivalency of the SHILO MR signal to the original 2D spoiled gradient recalled echo (SPGR) acquisition and its adequacy in sampling the AIF and tissue signals. Finally, we show feasibility of acquiring and analyzing SHILO DCE-MR images in human subjects with carotid atherosclerosis.

## Methods

### SHILO imaging sequence

**Figure 1:**
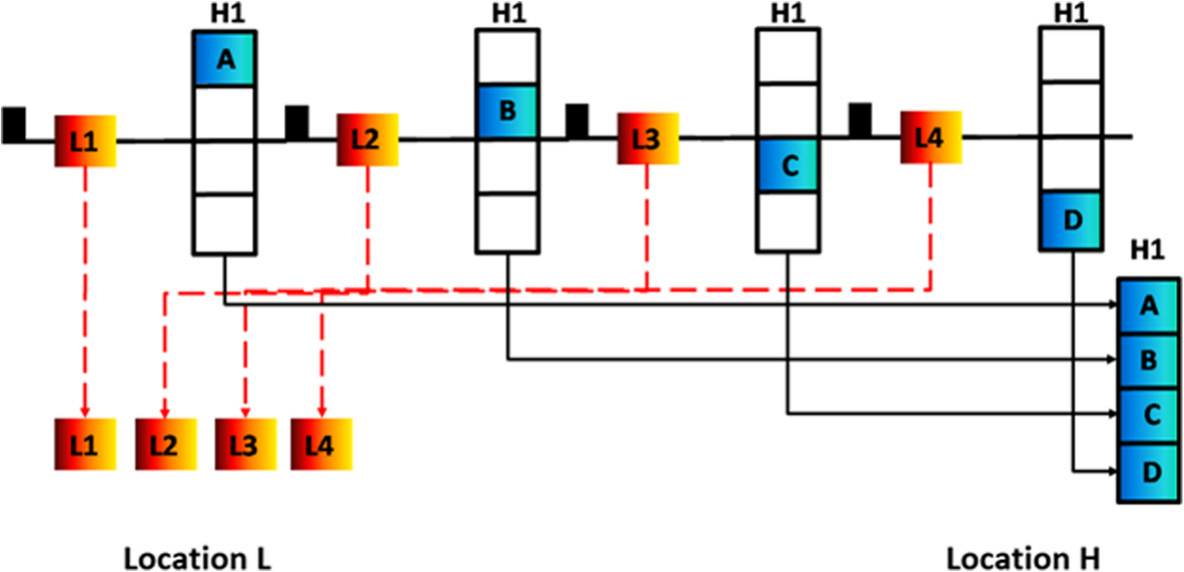
**Schematic representation of the SHILO segmentation/interleaving scheme.** The diagram illustrates an example where the high spatial resolution image is acquired in 4 segments, thus leading to the acquisition of 4 single shot AIF images for each segmented high resolution image. L1-4, single shot AIF images. A-D, segments of the multi-shot tissue image. H1: multi-shot high spatial resolution image coming from the combination of segments A-D. Low spatial resolution images L1-4 are acquired in location L, and high spatial resolution image H1 is acquired in independent location H. The black bar represents the saturation pulse preceding the low spatial resolution AIF image and high spatial resolution segment.

The SHILO sequence is based on a segmented, 2D, T1-weighted radio frequency (RF) spoiled gradient recalled echo (SPGR), turbo field echo (TFE) acquisition. The SHILO segmentation/interleaving scheme is shown in Figure 1. To allow for different time resolution between AIF and tissue acquisitions, the AIF is acquired as a single shot low spatial/high temporal resolution image (Figure 1, L1-4) while the tissue is simultaneously acquired as a high spatial/low temporal resolution multi-shot image (Figure 1, H1A-D). Therefore complete AIF frames are interleaved with tissue image segments. Spreading the acquisition of the tissue image over *n* segments, allows the time resolution of the single shot AIF image to be (*n* + 1)/2 times faster than if the tissue image was also acquired as single-shot, as in previously proposed dual-imaging schemes [15,37]. SHILO imaging parameters can be found in Table 1. Acquisition time for one segment is 0.8 s (TR × TFE factor). One AIF image is acquired every 1.6 sec, while one complete tissue image is acquired every 6.4 sec. AIF and tissue slices can be placed independently, and were typically separated by 15–20 mm in this case. A non-selective saturation pulse is played before every single-shot AIF acquisition (Figure 1, black bar). Similar to Kim et. al [15], to allow for different image contrast and dynamic signal range between AIF and tissue acquisition, a short saturation delay, *τ* = 0 ms, was used for AIF acquisition for increased sensitivity to the high concentration in the vessel lumen, while a longer delay, *τ* = 0.8 s, was used for tissue acquisition to allow for increased signal recovery before imaging. A repetition time (TR) of 10 ms was chosen to trade-off increased temporal resolution at lower TR with decreased signal saturation of longer T1 signal in the vessel wall at longer TR. Linear k-space reordering and a low flip angle of 15° was used for the AIF acquisition, similarly to Gatehouse et al. [37]. For the tissue acquisition, centric reordering was chosen so that contrast depends on recovery since the preceding saturation pulse rather than SPGR contrast. A higher flip angle of 25° was chosen to increase the signal excited at the center of k-space from the prepared magnetization.

**Table 1:** SHILO imaging parameters

**Imaging parameters**	**AIF**	**VW**
Field of view (FOV)	160 mm × 160 mm	
In-plane spatial resolution	0.5 mm × 2 mm	0.5 mm × 0.5 mm
N of slices	1	
Slice Thickness	3 mm	
Acquisition matrix	320 × 80	320 × 320
N of acquisition segments	1	4
Turbo field echo (TFE) factor	80	
Repetition time (TR)	10 ms	
Echo time (TE)	4 ms	
Saturation delay	None	800 ms
Flip angle	15	25
K-space	linear	centric
Time resolution	1.6 s	6.4 s

### Dual imaging with SHILO: temporal sampling simulations

Here we show that accuracy and precision of kinetic parameters related to atherosclerotic plaque microvascularization can be improved by sampling the AIF at faster time resolution than the plaque enhancement. This feature is implemented in the novel SHILO sequence by interleaving the low spatial/high temporal resolution AIF images with high spatial/low temporal resolution tissue acquisitions. A model AIF [43] with time resolution 0.1 s over a 5 minutes interval was used to compute tissue uptake curves using a modified Tofts-Kermode [44] model and a range of kinetic parameters, representative of vessel wall perfusion indices reported in the literature [45,46]. Kinetic parameters used in the simulation were *v*_*p*_, the fraction of intra-vascular volume; *K*^*trans*^, expressing the inflow of contrast agent from the plasma to the tissue compartment; *v*_*e*_, the fraction of extra-vascular extra-cellular space. The parameter *K*_*ep*_ expressing the backflow of contrast agent from the tissue to the plasma compartment was calculated as *K*^*trans*^/_*ve*_. 150 different combination of kinetic parameters were used, with *v*_*p*_ values 0.001, 0.005, 0.02, 0.05, 0.1, *K*^*trans*^ values 0.02, 0.06, 0.1, 0.15, 0.2 min^-1^, and *v*_*e*_ values 0.1, 0.2, 0.3, 0.4, 0.6, 0.8. Two different sampling schemes were then implemented while fitting for kinetic parameters [16]: 1) sampling of both AIF and tissue curves at the same increasingly lower rate (referred to as “same time resolution”, STR) to emulate deriving the AIF curve from the same tissue frames 2) sampling of the tissue curves only (referred to as “different time resolution”, DTR) at an increasingly lower rate to emulate the SHILO dual imaging or other dual bolus techniques. These different levels of under-sampling simulate situations in which an increasing number of tissue slices is acquired using both sampling schemes. In the STR scheme both AIF and tissue curves were under-sampled at rates 1.6, 3.2, 6.4, 12.8, 25.6 and 51.2 s (corresponding to multiples from 1 to 32 of the AIF time resolution in SHILO). In the DTR scheme, tissue curves were under-sampled at the same rate used for the STR case. As for AIF, two DTR schemes were investigated with the AIF sampled at 1.6 s and 0.8 s. The first case (1.6 s AIF sampling) corresponds to the temporal resolution of the AIF in the proposed SHILO acquisition. The second case correspond to a hypothetical test bolus acquisition, where the time resolution of the AIF is equal to the acquisition time of one single shot image as mentioned in the above description of the SHILO sequence, without interleaving the acquisition of segments of tissue slices. Comparing both these DTR schemes allows assessing the impact on kinetic parameters estimation of interleaving AIF and tissue acquisitions, such as is proposed in the SHILO sequence, as opposed to the corresponding test-bolus acquisition. For each parameter set and sampling scheme, the simulation was repeated 6 times, each time shifting the sampling grid by one sixth of the sampling interval to account for a variable temporal registration of the image sampling and bolus passage. Finally, kinetic parameters were estimated with the same modified Tofts-Kermode model used to simulate tissue curves [44], using non linear least squares fitting routines implemented in Matlab (MathWorks, Natick, MA). The average and standard deviation of estimated kinetic parameters across the 6 variable temporal registration cases were recorded. Relative errors, normalized by the true parameter value were recorded and the magnitude taken. The median of relative magnitude errors and standard deviation over the span of kinetic parameters simulated was plotted as a function of tissue slice temporal sampling resolution.

### Validation of SHILO imaging sequence: phantom experiments

The equivalency of the MR signal of the newly developed SHILO sequence with its low and high spatial resolution standard SPGR counterparts was investigated using phantom experiments. Ten vials containing different dilutions of Gd-DTPA in saline with concentrations between 0 to 5 mM were imaged using the SHILO acquisition and using standard saturation-prepared 2D-SPGR acquisitions equivalent to the single-shot low-resolution and 4-fold segmented high-resolution SHILO images. For the high resolution SPGR image a saturation delay of 0.8 s was used as in the SHILO acquisition. SHILO imaging parameters were the same as described above (Table 1). An additional proton-density-weighted image (PD-w) was acquired with the same imaging parameters described in Table 1 except TR/TE 300/2.3 ms and flip angle = 4° to normalize the signal. Regions-of-interest were drawn in each vial using custom made Matlab software (MathWorks, Natick, MA). Normalized signal from SHILO and SPGR images were plotted against each other and compared using Pearson’s correlation coefficient.

### Validation of SHILO imaging sequence: *in-vivo* imaging

**Figure 2:**
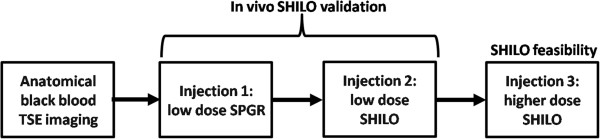
**Flow chart of *in vivo *CMR experiment.** After anatomical black blood imaging a low dose (0.01 mmol/Kg) 2D SPGR and a low dose (0.01 mmol/Kg) SHILO scans are acquired for *in vivo* validation of the SHILO MR signal. Following, a higher dose (0.05 mmol/Kg) SHILO acquisition was used to calculate perfusion parameters in the carotid vessel wall and sternocleidomastoid skeletal muscle.

In this experiment we verified that the AIF signal measured by the SHILO sequence was comparable to the AIF measured with an equivalent standard SPGR sequence *in vivo*. With approval from the Mount Sinai Institutional Review Board (IRB) and after written informed consent, four patients with either risk factors or history of atherosclerosis were recruited for carotid imaging with SHILO. Subjects were imaged on a 3T whole body scanner (Philips Achieva) using a dedicated 8-channel array for carotid imaging. After anatomical fast gradient echo and time-of-flight (TOF) angiographic scout images, standard T1, T2 and PD weighted turbo spin echo (TSE) black blood images were acquired for characterization of the vessel wall of both common carotid arteries using imaging parameters described in previous studies [47]. For normalization of the SHILO MR signal, an rf-spoiled 2D-SPGR image was acquired with TR/TE 300/2.3 ms and flip angle = 4° to give proton-density image weighting (PD-w) independent of T1 and T2 in each of the locations of the AIF and tissue uptake slices. AIF and tissue slices for SHILO DCE-CMR were then positioned in the common carotid arteries, perpendicular to the vessels. The tissue slice was positioned in the location with greatest vessel wall thickness as identified on multi-slice black blood TSE scans. The AIF slice was positioned 15–20 mm caudally with respect to the tissue slice. To confirm *in vivo* equivalency between the SHILO MR signal and the corresponding standard SPGR acquisition, subjects were injected twice, with a low dose of contrast agent, in the same imaging session. This approach allows minimizing the possible confounding effects of inter-scan variability. After placing an 18-gauge catheter in an antecubital vein, 0.01 mmol/kg of Gd-DTPA (Magnevist) was injected both times at a rate of 4 ml/s, followed by 20 ml saline flush (Figure 2). Contrast agent injection started after the acquisition of 3 high spatial resolution tissue images of the SHILO- DCE sequence (approximately 19.2 sec). During the first low dose injection, subjects were imaged using a low resolution SPGR sequence, equivalent to the AIF SHILO images. During the second low dose injection subjects were imaged using SHILO. Following acquisition, images were transferred to a workstation for image analysis. After image registration and de-noising [48] image signals were normalized by the PD-w images. One ROI was drawn in the vessel lumen of each common carotid for both the low resolution SHILO and SPGR AIF images. The average ROI signal intensity versus time was converted to T1 by using the SPGR signal equation, and converted to concentration of gadolinium in the blood [Gd]_blood_ using [Eq. 1]. Contrast agent relaxivity was assumed to be *r* = 4.2 s^-1^mM^-1^. The initial value of T_1,0_ was taken from the average of pre-contrast images before the influx of contrast agent. For kinetic analysis, the concentration [Gd]_blood_ was corrected for hematocrit.

(1) $${\left[\mathrm{Gd}\right]}_{\mathrm{blood}}=\frac{1}{r}\left(\frac{1}{T_{1}}-\frac{1}{T_{1,0}}\right)$$

Correspondence between AIF curves derived from SHILO and standard SPGR acquisitions was evaluated by visual inspection. AIF curves from all vessels of each subject were averaged after appropriate translation in time to overlay the initial bolus arrival. The Pearson’s correlation coefficient for this set of data points was found.

### Feasibility of SHILO imaging for carotid atherosclerosis

In the same cohort of patients, and in the same imaging session, after two low dose injections for *in vivo* validation of SHILO, an additional higher dose injection was used to assess feasibility of using SHILO for perfusion studies in carotid atherosclerosis (Figure 2). For this purpose, 0.05 mmol/kg of Gd-DTPA (Magnevist) was injected at a rate of 4 ml/s, followed by 20 ml saline flush. In this case also, contrast agent injection started after the acquisition of 3 high spatial resolution tissue images of the SHILO- DCE sequence (approximately 19.2 sec). Following acquisition, images were transferred to a workstation for image analysis. After image registration and de-noising [48] image signals were normalized by the PD-w images. One ROI was drawn in the vessel lumen of each common carotid for the low resolution AIF image, while for the high resolution tissue image ROIs encompassed the whole visible vessel wall of both common carotids and areas in the sternocleidomastoid muscle. The average ROI signal intensity versus time for both AIF and tissues was converted to T1 by using the SPGR signal equation, and converted to concentration using [Eq. 1]. Contrast agent relaxivity was assumed to be 4.2 s^-1^mM^-1^. The initial value of T_1,0_ was taken from the average of pre-contrast images before the influx of contrast agent. AIF concentration curves were corrected for hematocrit before kinetic modeling. A modified Tofts model [44] was used to analyze concentration curves, by using the AIF and tissue data from SHILO images. Kinetic parameters v_p_ (fraction of vascular space), K^trans^ (wash-in constant from plasma to tissue compartment), v_e_ (fraction of extravascular extracellular space), and K_ep_ (K^trans^/_ve_ wash-out constant from tissue to plasma compartment) were calculated using standard non-linear least squares fitting procedures [49] written in Matlab.

## Results

### Kinetic modeling simulations

**Figure 3:**
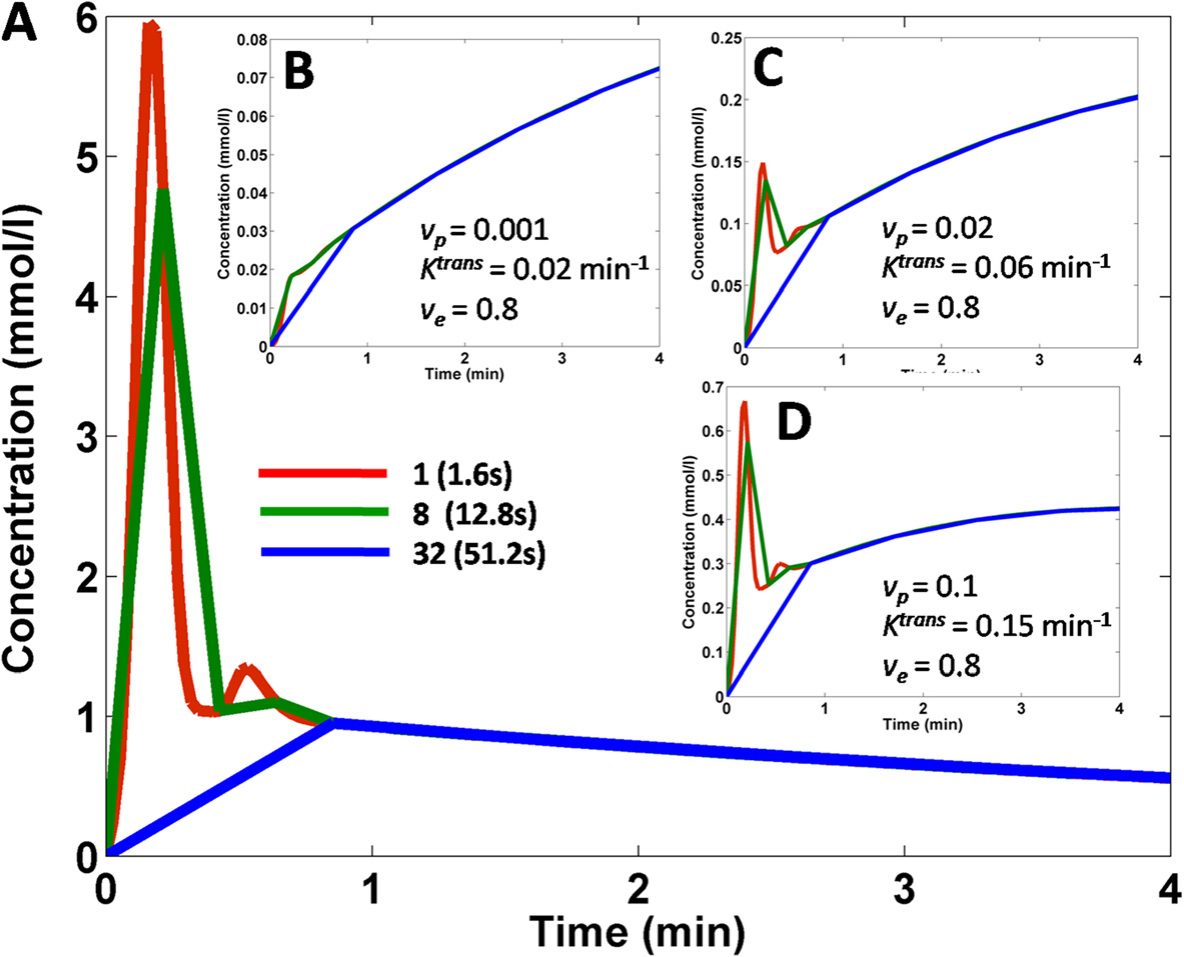
**Effect of under-sampling on simulated AIF (panel A) and three simulated tissue curves (panels B to D).** Different color curves represent different degrees of under-sampling, corresponding to decreasing time resolutions for an increasing number of imaged tissue slices, as detailed in the legend. Kinetic parameters used are specified in the legends.

**Figure 4:**
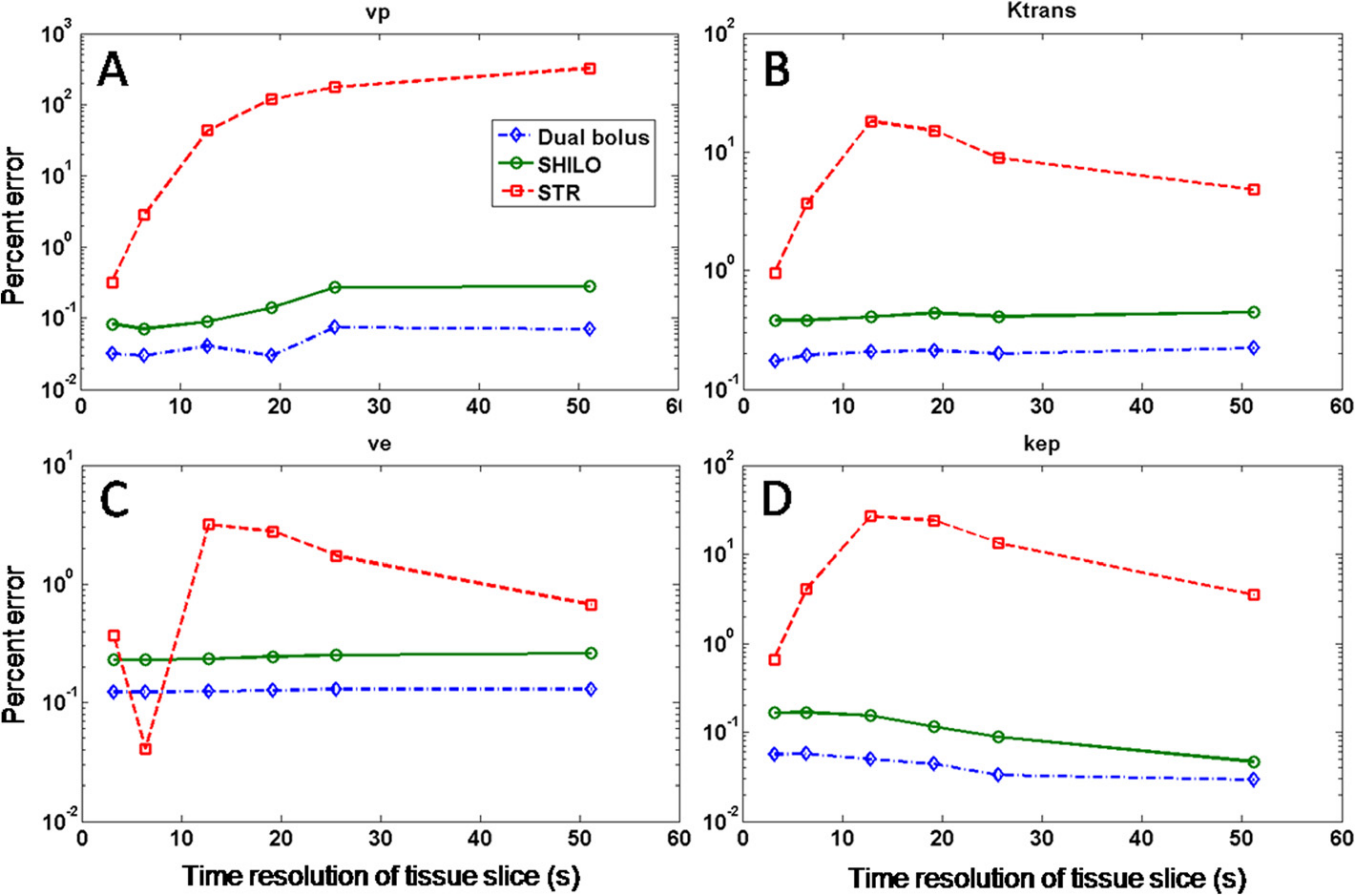
**Results of numerical simulations for percentage error.** Panel **A**, *v*_*p*_. Panel **B**, *K*^*trans*^. Panel **C**, *v*_*e*_. Panel **D**, *K*_*ep*_. Red dashed line, results for the same time resolution (STR) sampling scheme. Green solid line, results for the different time resolution (DTR) sampling scheme implemented in SHILO (AIF time resolution 1.6 s). Blue dash dotted line, results for the second different time resolution (DTR) sampling scheme with AIF time resolution 0.8 s.

**Figure 5:**
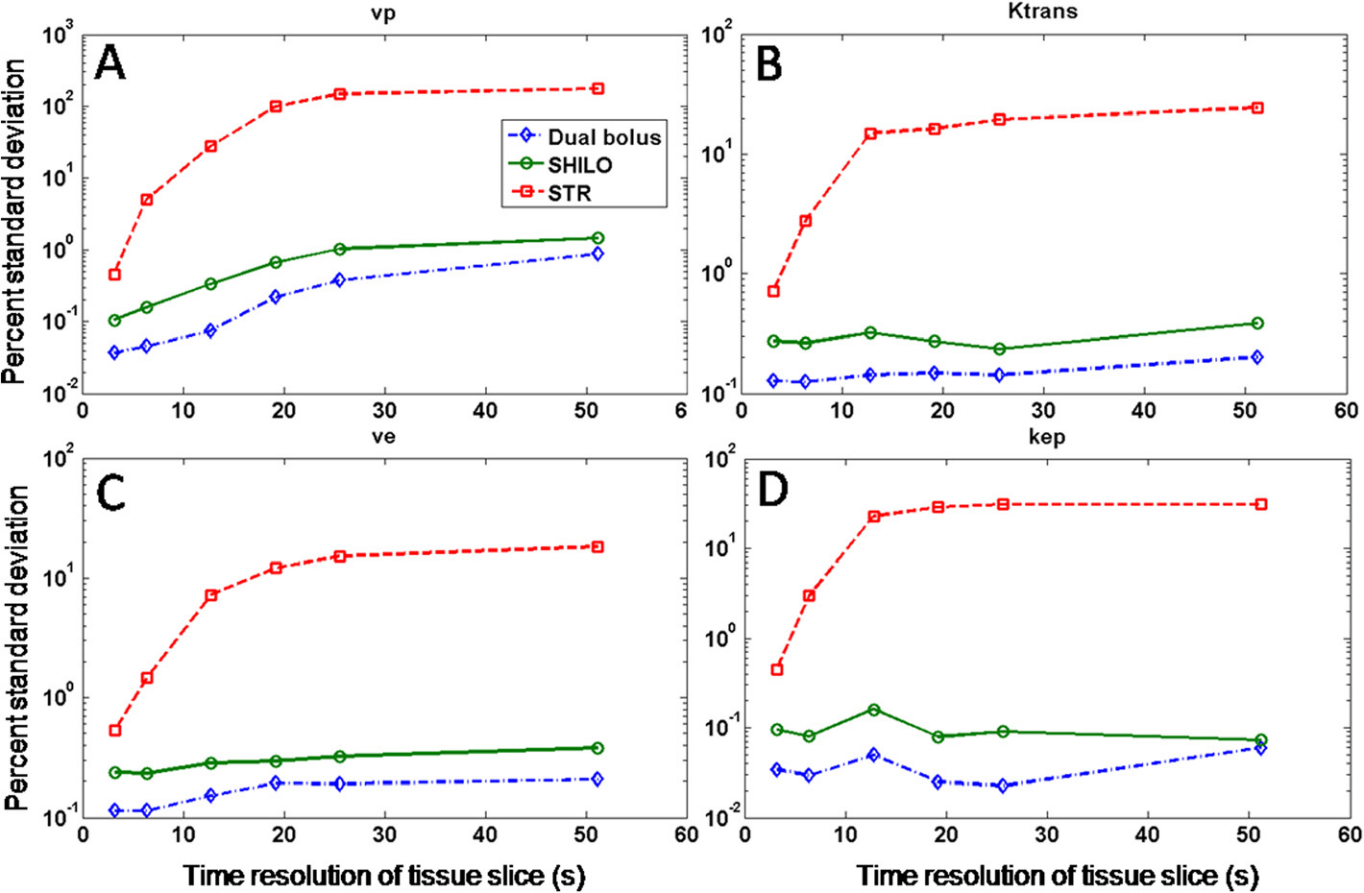
**Results of numerical simulations for percentage standard deviation.** Panel **A**, *v*_*p*_. Panel **B**, *K*^*trans*^. Panel **C**, *v*_*e*_. Panel **D**, *K*_*ep*_. Red dashed line, results for the same time resolution (STR) sampling scheme. Green solid line, results for the different time resolution (DTR) sampling scheme implemented in SHILO (AIF time resolution 1.6 s). Blue dash dotted line, results for the second different time resolution (DTR) sampling scheme with AIF time resolution 0.8 s.

Figure 3 shows the effect of under-sampling at different rates for the AIF curve (Figure 3, panel A) and three different tissue curves (Figure 3, panels B-D). Red solid lines represent the fully sampled data, while green and blue lines represent under-sampled data (8 and 32 times respectively). The plot demonstrates the dramatic effect of under-sampling on the amplitude of the AIF peak, as well as on the initial portion of fast enhancing curves (Figure 3, panels C and D). The effect of under-sampling on slower enhancing curves (Figure 3, panel B) is less evident. Results of the simulations are shown in Figures 4 and 5 for estimations of each of the kinetic parameters separately. Figure 4 shows the median of relative magnitude errors, while Figure 5 shows the median standard deviation over the span of kinetic parameters, plotted as a function of tissue curves sampling resolution. Errors and standard deviations for both DTR and STR schemes increase as the tissue temporal resolution is decreased, thus confirming the results of previous studies [16,17]. The log scale for relative error and standard deviation shows that DTR schemes vastly outperform the STR scheme, especially at lower temporal resolution (Figures 4 and 5). Also evident is the close similarity of the two DTR schemes with AIF temporal resolutions of 0.8 sec and 1.6 sec. This indicates that the penalty paid when interleaving AIF and tissue images in the SHILO sequence does not substantially affect the estimation of kinetic parameters with respect to the corresponding dual bolus acquisition. The accuracy and precision of kinetic parameter estimation with the DTR schemes was better than 1%. The median error, over the span of kinetic parameter sets simulated, for all tissue sampling rates was less than 1% (Figure 4). Similarly, the median standard deviation of the kinetic parameter estimation was less than 1% (Figure 5). Conversely, accuracy and precision of parameter estimation with the STR scheme was typically between 1% and 100%, as shown by the median errors and standard deviations shown in Figures 4 and 5.

### Validation of SHILO imaging sequence: phantom experiments

**Figure 6:**
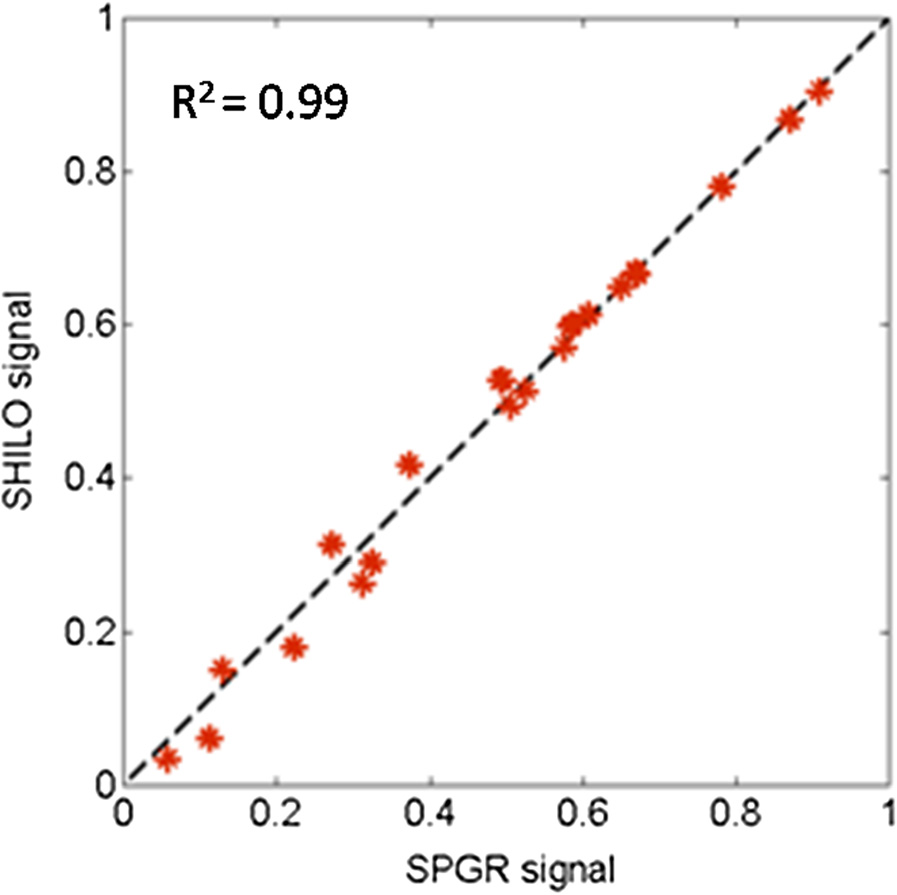
**Correlation of normalized signal values from SHILO and equivalent SPGR acquisitions in a phantom with a range of T1 values corresponding to gadolinium concentrations between 0 and 5 mM.** Correlation coefficient R^2^ = 0.99.

There is a close correspondence between SHILO and standard techniques for each of the comparisons: i) low-resolution SHILO (SHILO-LO) versus single-shot SPGR, and ii) high resolution SHILO (SHILO-HI) versus segmented SPGR with a 0.8 s saturation delay (Figure 6). Measured signal from SHILO is plotted against those from SPGR in Figure 6 and shows a correlation with R^2^ = 0.99. This indicates that the modifications required for interleaving AIF and tissue scans do not compromise the fidelity of signal acquisition required for accurate T1 measurements in the SHILO DCE scan.

### Validation of SHILO imaging sequence: *in vivo* experiments

**Figure 7:**
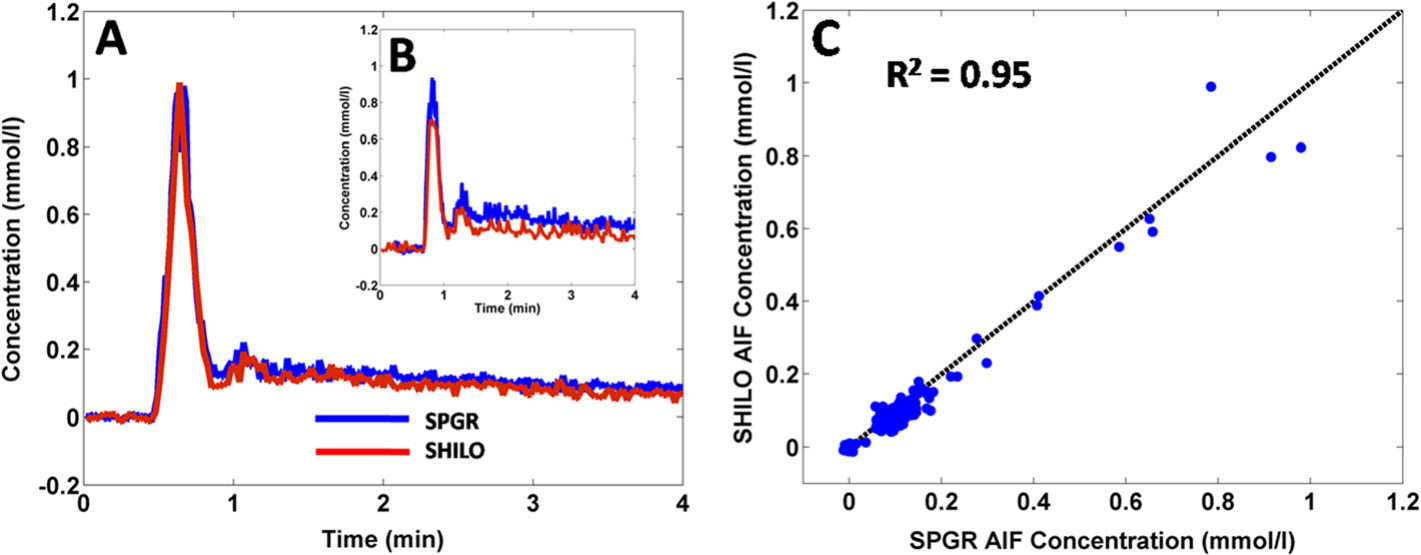
**AIF concentration-time curves measured using SHILO and an equivalent SPGR acquisition.** Panel **A** shows the average AIF for all vessels in all subjects. Panel **B** shows the AIF from one vessel in one subject. Panel **C** shows the correlation between the *in vivo* average SPGR and SHILO signals (R^2^ = 0.95; slope 0.95, intercept - 0.01).

There is a striking correspondence between the AIF shapes acquired with the SHILO sequence and the standard SPGR equivalent. Shown in Figure 7 are the AIF curves from SHILO and SPGR plotted on the same axes for (A) the average of all AIFs from all vessels in all subjects, and (B) for one single vessel. The correlation coefficient between the averages AIFs for SHILO and SPGR was R^2^ = 0.95 (slope 0.95, intercept - 0.01).

### *In-vivo* imaging of carotid atherosclerosis

**Figure 8:**
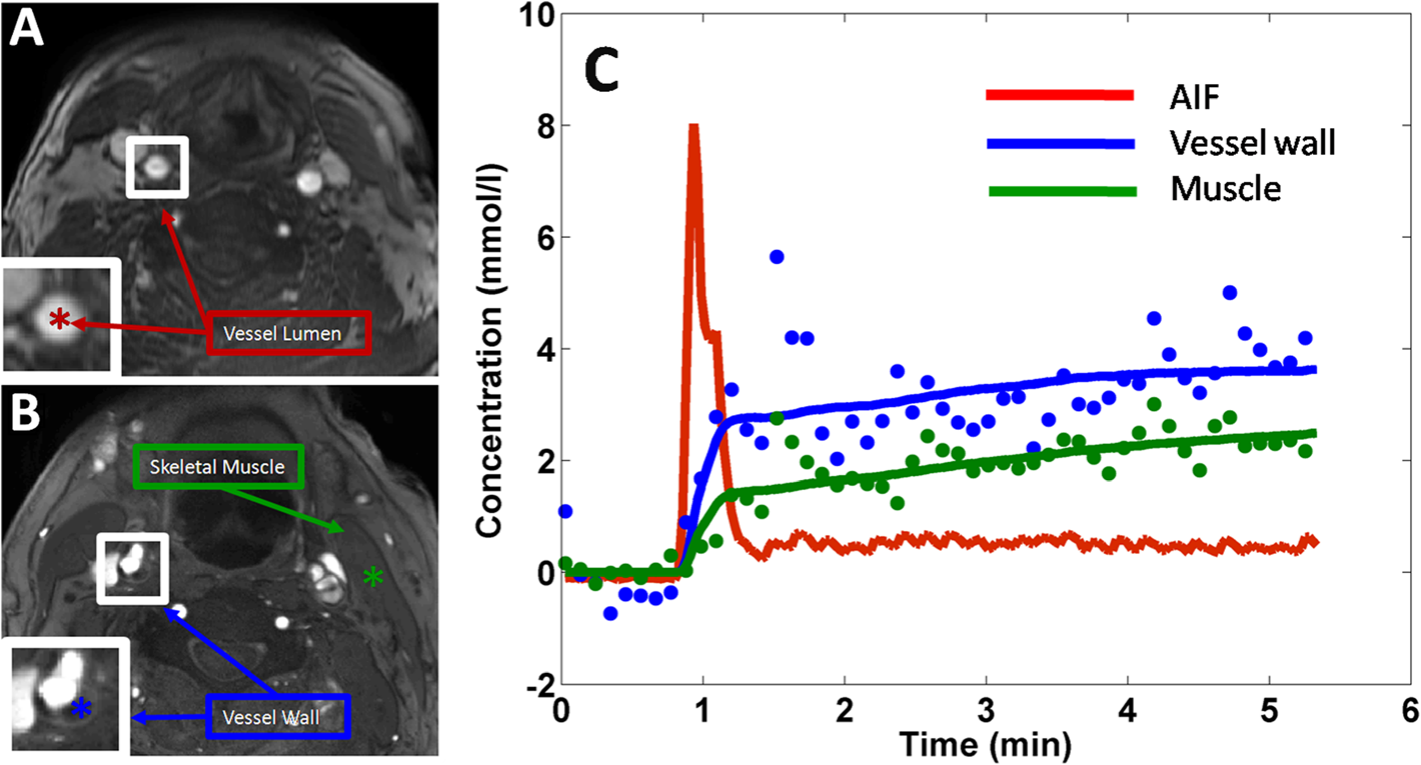
**Anatomical images of a representative subject, one axial slice and zoomed in insert on the right common carotid artery (bottom left of all panels).** Panel **A**, SHILO low spatial/high temporal resolution image for AIF acquisition. Red star in insert, ROI used to sample blood SI curve. Panel **B**, SHILO high spatial/low temporal resolution image for tissue data acquisition. In the insert, the ROIs used to sample the muscle (green) and vessel wall (blue) SI curves are depicted. Panel **C**: red line, AIF from low spatial/high temporal resolution SHILO; blue dots, vessel wall SI curve from high spatial/low temporal resolution SHILO; green dots, muscle SI curve from high spatial/low temporal resolution SHILO. Blue and green lines represent kinetic modeling fittings. X axis, time (min). Y axis, contrast agent concentration (mmol/l). Vessel wall and muscle curves are plotted 25 times their measured concentrations for clarity in the plot.

For a representative subject, AIF and tissue images taken from the SHILO sequence are shown in Figure 8A and B and indicate the image quality obtainable. Concentration-time curves for the AIF, vessel wall, and muscle measured from ROIs placed on the dynamic series of these images are plotted in Figure 8C. The AIF curve clearly shows the passage of the contrast agent bolus [43], and the tissue curves are consistent with shapes expected for low perfusion tissue [5,6,19,50-52]. Average kinetic parameter values for the vessel wall were respectively: K_trans_ : 0.27 ± 0.26 min^-1^; v_p:_ : 0.02 ± 0.03; v_e_: 0.53 ± 0.26 . Average kinetic parameters values for the skeletal muscle were respectively: K_trans_ : 0.09 ± 0.04; min^-1^ v_p:_ : 0.00 ± 0.00; v_e_: 0.27 ± 0.06. These values compare favorably with similar estimates found in the literature [6,51-53].

## Discussion

Abundant and permeable adventitial microvessels and intra-plaque neovascularization are considered histological hallmarks of plaque vulnerability [45,54] and therefore an attractive target for non-invasive imaging methods aimed at identifying individuals at high-risk for acute cardiovascular events [55,56]. DCE-MRI is a non-invasive technique used to quantify tissues’ perfusion in several organs [4], which has been applied in recent years to quantify perfusion in atherosclerotic plaques [5-8,50-52,57].

Accurate quantification of plaque perfusion is particularly challenging, because of the high in-plane spatial resolution and slice coverage required to capture the heterogeneity of highly complex plaques. Despite the initial encouraging results [5-9,48,50-52,57], current vascular DCE-CMR protocols used to assess plaque perfusion have failed to meet these requirements.

In this manuscript we propose a novel dual imaging technique for improved quantification of perfusion parameters for DCE-CMR of atherosclerosis. We name the new sequence SHILO (Simultaneous HI-/LOw-temporal (low-/hi-spatial) resolution DCE-imaging). The new SHILO sequence proposes to overcome challenges presented by the need to acquire both accurate AIF and tissue DCE curves simultaneously when high-spatial resolution and slice coverage are required for tissue acquisition, such as is the case of atherosclerotic plaques.

In this respect, the SHILO technique has several advantages over other dual imaging DCE techniques. The segmented nature of the tissue acquisition allows acquiring the tissue scan with higher spatial resolution than the AIF scan, while still maintaining a high temporal resolution on the order of 1 s for the AIF curve. In particular, segmentation allows simultaneous measurement of the AIF and the tissue response, allowing accurate sampling of the initial phases of both the plasma and tissue uptake curve (Figure 1).

Importantly, the AIF and tissue scans can be acquired with different dynamic signal ranges, accounting for the expected differences in contrast agent concentration in the lumen and tissue. Additionally, the separation of imaging segments makes the acquisition flexible, since imaging parameters can be manipulated to change signal contrast properties of either scan.

Finally, the AIF image can be acquired in a separate location from the main tissue scan as demonstrated here. This concept can furthermore be extended to multi-slice imaging of the tissue, while retaining a single slice for the AIF. This will allow adequate spatial resolution and slice coverage to capture the complexity of atherosclerotic plaques, while improving estimation of kinetic parameters because of more accurate AIF sampling and will be addressed in future work.

In this study we demonstrate the need for the dual imaging approach that is the basis of our proposed new acquisition sequence. Through numerical simulations, we quantify the errors that are likely to arise from DCE measurements if both AIF and VW wall enhancements are sampled at the same, slow, time resolution (STR sampling scheme). On the contrary, we demonstrate the efficacy of our proposed sampling scheme, in which the AIF is sampled with faster time resolution than the tissue (DTR sampling scheme). Relative errors and standard deviations for the DTR scheme were vastly lower than for the STR scheme, especially at lower temporal resolution (Figures 4 and 5). Also evident was the close similarity of the two DTR schemes with AIF temporal resolutions of 0.8 sec and 1.6 sec. This indicates that there is minimal penalty due to reducing the temporal resolution of the AIF to accommodate the tissue image segment in the interleaved SHILO DTR scheme. The median accuracy and precision of kinetic parameter estimation for the DTR schemes was found to be less than 1% while for STR sampling the accuracy and precision was between 1% and 100%. This may have significant impact on the estimation of plaque perfusion, since the indices *v*_*p*_ and *K*^*trans*^ have been previously shown to correlate with plaque neovascularization and several systemic risk factors in patients with carotid atherosclerosis [6,51].

The results of our numerical simulations are in concordance with similar studies presented by other groups in the field of perfusion imaging, which shows the robustness of this approach [16,17].

Following, we show equivalency of the SHILO MR signal to the signal of corresponding low and high resolution standard SPGR sequences (Figures 6 and 7). We demonstrate with both *in vitro* and *in vivo* experiments that the modifications applied to the native SPGR sequence to implement the SHILO sampling scheme, do not affect the properties of the SPGR MR signal. This is important when dealing with quantitative MR imaging techniques, such as DCE-CMR, where the signal has to be appropriately converted to concentration to extract meaningful quantitative information.

Finally, we show feasibility of using SHILO for DCE-CMR of subjects with carotid atherosclerosis (Figure 8). We demonstrate ability to extract meaningful kinetic parameters from SHILO acquisitions, in the same range of published literature values for both vessel wall and skeletal muscle, used as a reference tissue. In these early experiments we are encouraged by the comparison of kinetic parameters to those already in the literature. Rather than measuring substantially different kinetic parameters, we anticipate the benefit of SHILO to be in improved precision of kinetic parameter estimates that will improve the sensitivity of DCE measurements to neovascularization in the tissue. This will be investigated in further studies including correlation between DCE measurements and histological evaluation.

### Study limitations

This feasibility study presents several limitations. As shown by numerical simulations, some advantages of the DTR sampling scheme implemented in the SHILO sequence can be mostly appreciated when the disparity between the AIF and tissue time resolution is significant. This is the case when imaging more than one tissue slice, simultaneously with one AIF slice. In this feasibility study we omitted extensive exploration of comparison with acquisitions of multiple tissue slices, instead demonstrating the technique with a single tissue slice. Our aim was to demonstrate the feasibility of applying the DTR sampling scheme, while providing physical separation between AIF and tissue slices and independence of imaging parameters, attributes not available in other k-space sharing techniques. Comparison of tissue curves between SHILO and SPGR in the low dose scans was not possible, because of the too low uptake of contrast agent in the vessel wall. Addition of a second high-dose to make such a comparison would have increased total patient dose beyond the recommended guidelines. Finally, the small number of patients included and the lack of histological samples limits the extent of the assessment of the SHILO technique. However, we feel that the data presented in this feasibility study will warrant the future investigation of this novel approach in a more extensive study, where the absolute accuracy of plaque perfusion parameters will be compared against histological features as gold standard.

## Conclusions

In conclusion, the successful demonstration, *in vivo*, of the new SHILO dual-imaging technique for simultaneous AIF and tissue-curve imaging in DCE-CMR of atherosclerosis is promising, and warrants further investigation in wider studies measuring kinetic parameters of atherosclerotic plaque neovascularization.

## Competing interests

The authors declare that they have no competing interests.

## Authors’ contribution

CC conceived the new SHILO sequence, performed numerical simulations, phantom and in vivo experiments, and was responsible for preparing the manuscript and figures. PMR helped performing nmerical simulations, phantom and in vivo experiments, and provided critical review of the manuscript. SR, VM and MKT helped conceiving the new SHILO sequence, and provided critical review of the manuscript. MC helped with in vivo experiments and provided critical review of the manuscript. SEF programmed the new SHILO sequence and supervised the initial phases of the project and planning of phantom and in vitro experiments. ZAF provided supervision of the whole project and critical review of the manuscript. All authors, except SEF, read and approved of the manuscript. Despite his premature passing on June 28th 2011, SEF greatly contributed to this study.
